# Exploring work motivation in High and Intensive Care wards with the confession booth: An innovative approach

**DOI:** 10.1016/j.ijnsa.2026.100530

**Published:** 2026-04-16

**Authors:** Isa C. de Jong, Laura A. van Melle, Cornelis L. Mulder, Mariëtte A. van den Hoven, Yolande Voskes

**Affiliations:** a Department of Ethics, Law and Humanities, Amsterdam University Medical Centers, location VUmc F-wing Room F038 De Boelelaan 1089a, 1081HV Amsterdam, the Netherlands; b GGZ Ingeest, Amerikaweg 2, 2035 RA, Haarlem, the Netherlands; c Department of Psychiatry, Epidemiological and Social Psychiatric Research institute, Erasmus University Medical Center, Dr. Molewaterplein 40, 3015 GD, Rotterdam, the Netherlands; d Parnassia Psychiatric Institute, Prins Constantijnweg 131, 3066 TA, Rotterdam, the Netherlands; e Tilburg School of Social and Behavioral Sciences, Tranzo Scientific Center for Care and Welfare, Tilburg University, Simon Building, Professor Cobbenhagenlaan, 5037 DB, Tilburg, the Netherlands; f Mental Healthcare Centre GGZ Eindhoven, Dr. Poletlaan 40, 5626 ND, Eindhoven, the Netherlands

**Keywords:** Staff shortage, High and intensive care, Acute psychiatry, Staff motivation, Retention, Recruitment, Confession booth, Job satisfaction

## Abstract

**Background:**

In the Netherlands, the High and Intensive Care model has been developed to enhance the quality of care and reduce coercion on acute psychiatric wards, with staff recognized as a crucial factor in its successful implementation. However, high staff turnover threatens both sustainable implementation of the model and the well-being of patients and healthcare professionals.

**Objectives:**

To inform strategies for staff retention and recruitment, this study explores the motivations of professionals working on High and Intensive Care wards.

**Methods:**

We used an innovative arts-based method known as the 'confession booth' to collect data that uncovers deep motivations and encourages individuals to reflect anonymously. The confession booth, a designated room on the ward, invited staff to send voice messages via WhatsApp, in response to three prompts: 1) What impressed them most during their shift 2) What made them most proud, and 3) What frustrated them most.

**Settings:**

The confession booth operated simultaneously across eight High and Intensive Care wards in the Netherlands over the course of two weeks.

**Results:**

Thematic analysis identified four overarching themes, each comprising multiple subthemes: meaningful engagement, professional agency, collaborative competence, and security. Participants emphasized that High and Intensive Care work is driven by a deep sense of compassion and a strong commitment to supporting patient recovery. The complexity of the work, along with active involvement in clinical decision-making, was described as both meaningful and professionally rewarding. Effective teamwork, particularly within a flexible, skilled, familiar team, was seen as essential to both job satisfaction and safety. Feeling safe emerged as a crucial prerequisite for all aspects of the work in which effective collaboration and adequate support from law enforcement were essential to maintaining this safety.

**Conclusion:**

To safeguard care quality, the persistent vicious cycle of insufficient attention to staff retention must be broken through structural investment in well-being, recognition, and reward. Retention is shaped by meaningful work, opportunities to apply expertise, effective teamwork, and safe working conditions. The confession booth provided a flexible and anonymous space for reflection, offering valuable, though not unlimited, insights. These findings present a strong foundation for improving retention and recruitment within High and Intensive Care settings.

**Registration number:**

The Amsterdam Public Health Research institute embedded this study with reference number SQC20222–047 on August 2, 2022.

**What is already known about this topic?**

• High and Intensive Care work is complex and requires highly skilled staff to ensure effective implementation of the model and high quality care.
• High staff turnover and workforce shortages jeopardize the quality of care and service continuity.
• Contributing factors to staff turnover include inadequate salaries and training bursaries, low job satisfaction, high patient acuity, high assault rates, and insufficient managerial support.

Alt-text: Unlabelled box dummy alt text

**What this paper adds**

• Professionals are primarily motivated to work on HIC wards because of meaningful engagement, professional agency, resilient collaboration and security.
• Key recommendations include: implementing active recovery policies, enhancing clinician support, ensuring better compliance with High and Intensive Care admission criteria, increasing staff involvement in decision-making, improving salaries, preventing team overload, strengthening team-building efforts, prioritizing training and professional development, fostering closer collaboration with law enforcement and advocating for decisive action from legal authorities.
• To retain a skilled workforce, institutions must proactively break the vicious cycle of staff turnover by aligning organizational strategies with the intrinsic motivations of their staff, recognizing that the team is the cornerstone of effective HIC care.

Alt-text: Unlabelled box dummy alt text

## 1 Introduction

This study focuses on High and Intensive Care wards in psychiatric institutions in the Netherlands. Since 2013, all acute psychiatric wards in the Netherlands work with the High and Intensive Care model, which consists of best- and evidence based practices that are in line with care ethics (Voskes et al., 2021a) and recovery oriented care (Anthony, 1993). The model was developed to provide optimal short-term admission for people in severe psychiatric crisis and for whom outpatient care is temporarily insufficient. During High and Intensive Care admission, the aim is to use the least amount of coercion, in particular seclusion, and focusing on a strong patient-caregiver relationship and providing care through meaningful connections.

High and Intensive Care is a stepped care model, meaning it contains strategies to intensify levels of care, based on the patients’ needs: from a group based high care unit to an intensive care unit if a patient needs one-to-one care in a separate area, to a high-security room used as a last resort for emergency situations unmanageable outside the high-security room (Voskes et al., 2021b). The intensive care unit is an individual space with private sanitation and a bedroom where patients in crisis can temporarily stay accompanied by a nurse or family. The high-security room is a confined area with private sanitation and smart home technology to maximize the patient’s control over their surroundings. Its use constitutes a coercive intervention that must be continuously supervised and applied for the shortest time possible. Alongside the High and Intensive Care model, a High and Intensive Care monitor has been developed and validated, which is used to conduct audits to assess compliance to the model (van Melle et al., 2019).

The High and Intensive Care model qualifies as a 'complex intervention' because it integrates a diverse set of components, blending traditional inpatient treatment with recovery-oriented practices (Voskes et al., 2021b). Staff are a key factor in the successful implementation of the High and Intensive Care model and in reducing the use of coercive measures, for two main reasons. First, the admission experience of patients strongly depends on the quality and continuity of their interactions with healthcare professionals. Ensuring sufficient and competent staff is essential to provide the attention and therapeutic engagement each patient requires. Second, the care team plays a critical role in fostering both safety and recovery. The team’s ability to work collaboratively directly influences the extent to which a safe, supportive, and recovery-oriented environment can be established and maintained (van Melle et al., 2021a). However, High and Intensive Care wards are faced with high staff turnover and struggle to maintain stable teams, jeopardizing their ability to uphold the High and Intensive Care model's standards (van Melle et al., 2021b).

This challenge reflects a broader issue within the national mental health care system, where rising demand for services coincides with years of inadequate funding and insufficient reimbursement from insurance companies and government programs (Launder, 2020). At the same time, long waiting lists, increasingly complex patient needs, and a significant escalation in staff shortages place mounting pressure on the entire sector (Butryn et al., 2017; Adams et al., 2021; O’Dowd, 2024). High staff turnover and shortages typically reduce care quality and staff and patients’ well-being. Staff turnover and shortages have shown to increase the risk of treatment errors and the use of compulsory interventions, to threaten patient safety and to inflict harm and re-traumatization upon patients (Baker et al., 2019; O'brien-Pallas et al., 2010; O’Dowd, 2024). While some studies have shown positive effects from staff turnover, such as retrieving fresh perspectives, new ideas, and valuable experiences, the continuous need for recruitment, orientation, and training of new staff place a significant economic and non-economic burden on the system as well (Adams et al., 2021; Johnson et al., 2018). The complex nature of acute psychiatric care itself makes recruitment especially challenging, as it requires highly specialized skills and competencies that do not appeal to all professionals (Deacon et al., 2006). Several studies have examined staff turnover and found that low salaries and training bursaries (UNISON, 2017), low job satisfaction (Butryn et al., 2017), high acuity, high assault rates, and a lack of managerial support (Ward, 2011) cause people to leave their jobs. The lack of clinicians in psychiatry is a worldwide problem and often attributed to a lack of training positions and funding (Aggarwal et al., 2022), compounded by the aging workforce and a growing trend toward part-time hours (Adams et al., 2021).

While staffing issues require a strategic approach, the question remains which measures can sustainably enhance workforce stability and recruitment. It is widely recognized that recruitment strategies are ultimately ineffective when retention issues are not properly addressed (Adams et al., 2021). Also, given the scarcity of resources, it is essential to implement the most effective and appropriate recruitment strategies. Given the crucial role of staff in High and Intensive Care, understanding what motivates and challenges professionals is essential for developing effective recruitment and retention strategies. Therefore, the research question of this study is: what motivates professionals to work on a High and Intensive Care ward, and how can these motivations be effectively leveraged to retain and attract staff?

To study this issue, we use an innovative method called ‘the confession booth’ which is an arts-based method that was inspired by an educational project in the United Kingdom where anonymous confessions were collected about adolescents’ personal and professional failures (Cox, 2015). The confession booth was considered cathartic and much needed to raise awareness on certain topics and open a dialogue. By applying this method, we aim to create a space where people feel free to talk about their motivation working at a High and Intensive Care without intervention of others. Moreover, we aim to capture 'real' and authentic responses, based on daily examples and to enhance reflexivity (Ash et al., 2024).

## 2 Methods

### 2.1 Design

This qualitative study employs an arts-based methodological design, referred to as ‘the confession booth’ wherein participants share their experiences by sending voice messages.

### 2.2 Confession booth

‘The confession booth’ in our context is an arts-based method that is used to anonymously collect stories of people stimulating self-expression and reflection without judgment. Our method was inspired by initiatives in England, where master students in creative and cultural entrepreneurship used a confession booth to raise awareness and foster dialogue around adolescents’ personal and professional failures (Cox, 2015). The students collected anonymous confessions online, in start-up hubs, and via an old telephone booth outside a university. Participants described the experience as cathartic and much needed. This initiative inspired subsequent projects, including the National Health Service’s ‘100 Voices’ art project in hospitals (Sound Voice, 2025) and the appearance of confession booths at festivals as forms of artistic expression and entertainment (Purity Productions, 2025; Geisler, 2024; Compass Presents, 2025). Across these contexts, the confession booth has served multiple purposes: celebrating individuality and inclusion, inspiring creative work, providing a cathartic space for expression, amplifying rarely heard voices, and introducing moments of reflection and fun (Sound Voice, 2025; Purity Productions, 2025; Geisler, 2024; Compass Presents, 2025).

The confession booth, tailored to the specific High and Intensive Care context, was selected for this study as it provided an anonymous and engaging way to elicit first-hand reflections from clinical practice, minimizing external influence. By applying this method, we intended to lower social desirability bias which occurs when respondents give answers to questions that they believe will make them look good to others, concealing their true opinions or experiences (Hecker and Kalpokas, 2025). The confession booth was deemed particularly appropriate in this setting, as it provided a dedicated environment for real-time reflection, thus mitigating the risk of recall bias (Hecker and Kalpokas, 2025). This function was especially valuable within the High and Intensive Care context, where the rapid pace and demands of daily work can often obscure meaningful experiences and decision-making processes.

### 2.3 Wards and participants

A total of 24 mental health institutions, which participated in a larger High and Intensive Care study, were invited to participate in this study. Due to resource constraints, participation was limited to a maximum of ten institutions. Ultimately, eight institutions consented to participate in the research. Each ward appointed a contact person who was responsible for coordinating the project locally and for maintaining communication with the research team. To inform and recruit participants, large posters were displayed at the wards, and a small incentive in the form of a treat was provided. The study was intended for all staff members of the High and Intensive Care. Accordingly, all staff members were informed about the study via email as well as through printed materials on the ward.

### 2.4 Data collection

Before the main study, a brief pilot study was conducted at the authors’ own research department for colleagues, to test and refine the methodology and techniques used. Afterwards, at each High and Intensive Care ward, a dedicated space was set up for the confession booth for a two-week period. The confession booth was accessible 24/7, accommodating the realities of shift-based work. Upon entry, participants encountered three posters with the following prompts: In this shift 1) I was most impressed by 2) I was most proud of, and 3) I was most frustrated by. In the absence of observers, participants were free to select and respond to any or all prompt(s) and send their voice message(s) via WhatsApp by scanning a Quick-Response code. The prompts were designed to guide participants in sharing experiences and generating examples related to their motivational drivers encountered during their shift. No restrictions were imposed on the number of voice messages per participant.

### 2.5 Analysis

A thematic analysis was conducted following six phases outlined by Braun and Clarke as described in Gray (2018): (1) familiarize yourself with the data, (2) generate initial codes, (3) search for themes, (4) review themes, (5) define and name the themes and (6) produce the report. In phase 1, the voice messages were retrieved from WhatsApp and directly transcribed with the Microsoft Word Transcribe function to ensure de-identification. The first author read the manuscript and noted down initial ideas. In phase 2, the data was independently coded by author 1 and 5, using VERBI Software (2021), employing the three prompts (frustration, pride and being impressed). The researchers compared their codes and reached consensus through discussion. In phase 3, overlapping codes across categories were identified, and codes were merged where appropriate. This process led to the eventual abandonment of the initial categorical framework, with ongoing reference to the central research question: what are the motivations of care professionals to work in a High and Intensive Care setting? Phase 4 involved a collaborative session between author 1 and 2 to explore potential overarching themes, resulting in the identification of eight preliminary main themes and three minor themes. In phase 5, author 1 and 5 further refined the thematic structure, consolidating the preliminary themes into five overarching themes and ten subthemes, which were then defined and named. Finally, in phase 6, representative data extracts were selected to illustrate and substantiate the identified themes.

### 2.6 Ethical approval and informed consent

The Medical Ethics Review Committee of the Amsterdam University Medical Centers declared that this study is a non-Law Medical-Research study with people [niet-WMO] with reference number 2022.0625 (VU, 2022). The research institute embedded this study with reference number SQC20222–047.

We followed the ethical guidelines stated in Elsevier's Publishing Ethics Policy. This study was conducted and reported in line with the Standards for Reporting Qualitative Research (SRQR) and the IJNS & IJNS Advances author checklist. Two authors were employed at two participating High and Intensive Care institutions; however, they were not part of the High and Intensive Care team involved in the study. Their roles at the institution did not influence the data collection process or the interactions with the participants.

All participants were informed of the study's purpose and their voluntary participation in written information about the study and that by sending a voice message, participants agreed to participate in the study. To ensure participant privacy, multiple measures were implemented. First, a research WhatsApp account was created on a separate work phone used exclusively for research activities and managed by author 1. Second, incoming messages from participants were tracked solely based on their date, time and quantity. Third, participants were able to delete any voice messages they had sent in case they wanted to correct their message or withdraw from participation. Fourth, the messages were transcribed with transcription software to de-identify the voices. Fifth, voice messages were not listened to nor were phone numbers stored. Only in case of errors in the transcript, author 1 would listen back to the concerning audio file to correct the transcription. Sixth, all messages were deleted from the research WhatsApp account, and participants were notified accordingly.

## 3 Results

Over a two-week period, a total of 54 confessions were collected from 47 professionals, with each confession representing a single moment in time. One participant deleted their entire message. Participants made 47 confessions related to frustration, 42 related to pride, and 27 expressing a sense of being impressed (see Table 1). Most confessions were done during the day shift (51%), followed by the evening shift (40%) and the night shift (9%). As outlined in the ‘analysis’ section, the initial framework based on the original prompts (frustration, pride and being impressed) was ultimately set aside to facilitate an open thematic analysis. This process led to the identification of four overarching themes: meaningful engagement, professional agency, collaborative competence and security; each with corresponding subthemes (see Table 2).

**Table 1:** Frequencies of confessions per prompt.

Prompt	Frequency
In this shift, I was most impressed by	27
In this shift, I was most proud of	42
In this shift, I was most frustrated by	47

**Table 2:** Themes and subthemes from the analysis.

Themes	Subthemes
Meaningful engagement	Compassion Relational recovery
Professional agency	High and Intensive Care-compliant care Participation in decision-making Recognition and fair compensation
Collaborative competence	Resilience and flexibility Familiarity and trust
Security	Psychological safety Legal support

### 3.1 Meaningful engagement

It became clear that working at the High and Intensive Care had an impact on participants which they described either as impressive or as a source of pride. Participants felt compassion for their patients and the clinical conditions they were in. Participants reported feeling deeply moved and expressed additional pride when they were able to establish contact with their patients and contribute to their recovery. At the same time, participants were aware of the mandatory nature of the High and Intensive Care ward and were proud when they succeeded in preventing or using the least intrusive coercive measure.

#### 3.1.1 Compassion

Participants were impressed by the complexity of mental health conditions patients experienced, as well as by the resilience and perseverance patients showed in coping with these challenges. On occasion, participants reported being emotionally affected, particularly in cases where they shared demographic characteristics (e.g., age) with the patients. Moreover, participants expressed compassion toward patients who exhibited fear during the administration of coercive interventions, such as forced medication or seclusion, and reported being moved by the patients’ inherent vulnerability. Although some situations were described as confronting and sad, participants reported feelings of empathy and admiration toward their patients. They recognized them fundamentally as human beings affected by illness, something that could happen to anyone.

*“The most impactful experience for me was seeing a very young boy who was severely disturbed, suffering from severe catatonia. His parents were desperate, and the boy was exhausted from fear. What strikes me as so profound is that someone so young, with their whole life ahead of them, can reach a point where they become completely stuck. On one hand, this is confronting and deeply sad, but on the other hand, I find it incredibly valuable that I can contribute to his recovery, hoping that he will soon be able to pick up his life again.”*

#### 3.1.2 Relational recovery

Participants expressed considerable pride when they succeeded in establishing contact with patients, especially when it was difficult. Making contact required a certain level of mutual trust, which helped professionals build rapport and fulfil their clinical responsibilities. Participants clearly derived satisfaction from being able to attend to all their patients during their shift and frequently mentioned their effective use of the ‘first five minutes at admission’, which aims to improve contact early on. This made them particularly proud, especially in light of the struggles patients had often encountered with the police or the ambulance services prior to admission. Participants valued the rare opportunity to engage with patients during deeply personal and vulnerable moments. They felt grateful to be able to contribute meaningfully to the recovery process, offering support and presence when it mattered most.

*“I am specifically thinking of a situation where a young woman returns to her apartment. As she steps inside, the signs of her crisis and decompensation are immediately clear. The house is completely in disarray, with fear and distrust visibly manifested in the surroundings. You stand beside someone who is increasingly aware of this reality. The trust you receive as a nurse to gain insight into such a personal and confidential situation is remarkable. It feels quite intimate, does it not? And yet, to work together towards recovery, supporting and coaching the patient through that process, is always something that remains deeply impressive.”*

Good contact was also important to prevent coercion. Participants expressed considerable satisfaction about their ability to prevent the use of coercion or to use less intrusive coercive measures such as the transfer from the high-security room to the intensive care unit. Participants demonstrated a strong awareness of the mandatory nature of the High and Intensive Care ward, and emphasised their commitment to strive to reduce and prevent coercion as much as possible. For participants it felt very rewarding and valuable to observe results from their work, particularly, when they witnessed improvements in the patient’s condition, or when patients explicitly communicated signs of progress, even when this happened in small steps.

*“I am most proud of how, together with my team, I can make a difference in people's lives. Going back to the core reason why I chose this profession in the first place. That is the most rewarding thing you can do.”*

### 3.2 Professional agency

Participants made a strong appeal for applying their expertise as trained and experienced professionals at a highly demanding context of the ward. They expressed pride of doing High and Intensive Care-compliant work and they were frustrated when their professional input was excluded from the decision-making process. They wanted to feel recognized for the complex work they do, both in working conditions and in salary, which represented key points of frustration.

#### 3.2.1 High and intensive care-compliant care

Participants were keen on working according to the High and Intensive Care model and emphasized the importance of all staff members, including physicians, to uphold these standards. Deviations from the model were perceived as suboptimal care and were described as demotivating. They appreciated complex and demanding work, as it gave them a sense of fulfilment—allowing them to do what they were good at and what they were trained for. Therefore, they were frustrated over administrative burdens that, though part of the High and Intensive Care work, kept them from direct patient care. Most of the frustration, however, was related to prolonged stays without clearly defined treatment objectives, due to failed transitions from the High and Intensive Care to other wards, e.g. to supported living.

*“It has been very quiet recently; there are many patients who require little care and, in my view, are not all High and Intensive Care-worthy, yet they remain here for a long time. That does not motivate me to bring out the best in myself, I find that disappointing, and it makes the work less enjoyable. I prefer having many seriously ill patients, where I can make a significant difference, but I am not experiencing that now.”*

#### 3.2.2 Participation in decision-making

Participants reported deriving satisfaction from being recognized for their expertise and from being able to apply this in patient care. This indicated a desire to be actively involved in clinical decision-making, particularly concerning medication type and dosage. Nevertheless, their opportunities to contribute were limited, which they found particularly frustrating when inexperienced physicians did not invite their input. Due to persistent staff shortages, many of these physicians were unfamiliar with the High and Intensive Care model, leading not only to less assertive and effective crisis management but also to disruptions in continuity of care. Participants strongly believed that their own insights could have helped reduce distress, both for the patient in crisis and for others in the ward, and ensured that care was still delivered in a responsible and safe manner. At the same time, they underscored that the emphasis on minimising coercion and implementing the High and Intensive Care model must not compromise patient or staff safety. They highlighted the importance of acknowledging their safety-related concerns and emphasized that physicians should respect the professional boundaries nurses establish in critical situations.

*“What was most frustrating was that the on-duty doctor did not listen to our boundaries, especially when we requested that a patient be admitted to the high-security room due to aggression right before the admission and a history of known aggression. Despite this, the doctor insisted on receiving the patient in the assessment room, even though we repeatedly stated our concerns, continuously trying to cross our boundaries. In the end, we stood our ground, and I am proud of that. However, it is disheartening when, for safety reasons, you set boundaries, and it feels as though someone just disregards them.”*

While patient autonomy was acknowledged as important, participants described working within this emphasis, especially as reinforced by the Compulsory Mental Health Care Act, as sometimes difficult in the context of acute care. As they observed that too much focus on patient autonomy resulted in patients deteriorating and inflicting harm upon themselves.

*"Working according to the Compulsory Mental Health Care Act contributes to staff turnover. This is due to the strong emphasis on the autonomy and independency of the client, even while they are in crisis. Knowledge or experience [of staff] is no longer important, as everything is supposed to be done in consultation, ideally in cooperation with the client, while in 9 out of 10 cases, the client has no insight into their illness or even awareness of it. This results in, for example, severely psychotic individuals being admitted voluntarily without clear treatment agreements. What happens then, or what is aimed for, is that things must go wrong first before appropriate action is taken.”*

To counterbalance this, participants advocated for greater professional control at the outset of the admission process, aiming to gradually restore autonomy and better prepare patients for discharge, particularly those presenting with suicidal or psychotic symptoms.

#### 3.2.3 Recognition and fair compensation

Whereas the previous themes illustrated predominantly positive experiences, this theme was characterized mainly by frustrations. Participants felt insufficiently recognized and rewarded for their complex work, which requires considerable expertise. Moreover, High and Intensive Care work involves significant responsibility and frequent exposure to aggression, factors for which they felt inadequately compensated, leading to frustration. A few participants expressed dissatisfaction with the stark contrast between their salaries and those of temporary staff, as well as psychiatrists, who were, unlike them, financially compensated for workforce shortages and high workload. Additionally, participants wished for more personal time, greater autonomy over their irregular work schedules to achieve a better work-life balance. One participant reported working 60 hours in one week to fill in the staff gaps, followed by periods without any scheduled shifts.

*“It is indeed very dynamic and intense work. A lot is asked of you, and you often work weekends, with irregular hours. Frequently, you are working alongside temporary staff and freelancers who earn much higher hourly rates than you, with far fewer responsibilities. And that really shouldn’t be the case. Those who commit to this department should be rewarded for that commitment, and by 'rewarded,' I mean financially. It should be financially attractive to work here. It is not that offering higher pay would bring in people who are not suited for the job.”*

### 3.3 Collaborative competence

Many participants praised the skills of their team and individual colleagues. Participants expressed pride in their colleagues’ resilience and flexibility in jointly completing their tasks and maintaining high standards of care, despite challenging working conditions. Moreover, participants found great satisfaction in working with experienced, familiar and knowledgeable colleagues. In contrast, unfamiliarity with the High and Intensive Care model often led to frustration and disrupted teamwork.

#### 3.3.1 Resilience and flexibility

Participants expressed a strong sense of pride in the resilience and adaptability demonstrated by both themselves and their colleagues. They described how various challenges, such as crowding, understaffing, and limited facilities, required a high degree of flexibility—including the need to cover shifts in other wards within the organization. Although this was challenging, participants expressed pride in successfully managing their shifts, collaborating effectively with colleagues, and delivering the care they envisioned, all while maintaining calmness on the ward. One participant expressed frustration that colleagues were unmotivated to engage in new activities, such as participating in research. At the same time, participants felt a sense of pride in the teams’ resilience and willingness to offer second chances to patients, despite adverse events during prior admissions.

*“Today, a complex and challenging patient is returning from a forensic setting, a patient with whom the team has already experienced a lot during previous admissions. And yet, they simply do it again — they can almost forget, I would say, though of course not entirely, what happened during the previous admissions, they put it behind them and stand up for this patient again. They want to welcome them back and give them a new chance. I really think that is the beauty and the strength of our High and Intensive Care team: the resilience, the ability to keep moving forward, to pick up and continue.”*

#### 3.3.2 Familiarity and trust

Participants expressed strong appreciation for truly knowing their team members and for their skills and expertise, emphasizing how familiarity fosters trust and confidence in collaborative work. Providing and receiving feedback was considered important for fostering effective teamwork. This made the collaboration feel pleasant and secure, whereas working with unfamiliar colleagues often led to discomfort and a strong sense of insecurity.

*“There was too little staff on another ward due to a lot of sickness, and someone from our team had to go and work there. No one likes that. No one enjoys it, because there we are working with very few and unfamiliar staff with the most intensive patients in this tower. I ended up going because I did not want to leave my colleagues in the lurch. I was really frustrated by it because it was a shift where I felt unsafe, both with the patient population and especially with the colleagues I was working with, whom I did not know and was unsure of what I could rely on.”*

While some participants appreciated getting to know new colleagues and seeing their enthusiasm for the High and Intensive Care model, others expressed frustration at having to repeatedly explain policies to inexperienced staff, due to a lack of time and resources for proper training and professional development. Participants were particularly concerned about freelance physicians whose unfamiliarity with the High and Intensive Care model frequently resulted in care practices that deviated from its principles and standards.

*“Many freelancers come to work as physicians who are not well informed about the High and Intensive Care methodology, where we work with a high-security room and try as much as possible not to use this. And I notice that due to the unfamiliarity with the High and Intensive Care model and the physicians currently working there, in my opinion the door closes more often than necessary.”*

### 3.4 Security

Security emerged as a central theme throughout the study, serving as a strong indicator of and prerequisite for factors contributing to job satisfaction. Participants frequently spoke positively about their sense of safety, but also mentioned negative aspects that made the work feel unsafe, which they attributed to multiple factors.

#### 3.4.1 Psychological safety

The sense of safety was identified by participants as a key factor motivating their work. They described several elements that contributed to feeling safe: the availability of an high-security room for highly aggressive patients, the use of medication to stabilize situations, the presence of experienced and familiar colleagues, a stable team with a positive feedback culture, a good ward climate and a balanced patient population. Conversely, several factors were perceived as compromising safety. These included malfunction of the location tracking for the panic button system, pregnant staff that worked in patient contact and doctors who did not respect nurses' professional boundaries when it came to safety issues.

*“In my opinion, there also needs to be a sense of safety — the feeling of being secure. People who have been working there longer tend to settle for less in terms of safety; they see themselves more as tools. Younger colleagues eventually break because of it. So ensuring more safety is really a basic condition that an institution must provide.”*

#### 3.4.2 Legal support

Participants expressed frustration regarding perceived injustice when legal charges were dismissed in cases of severe aggression, which they attributed to behaviour issues rather than solely to the patients’ clinical condition. They expressed a desire for more consistent legal accountability in instances of aggression toward staff, emphasizing that such incidents should not be excused purely on the basis of an individual’s mental health status. Another source of frustration was the perceived lack of support from the police. Participants felt that the police often failed to take their concerns seriously and were reluctant to assist them with aggressive patients.

*“I want to help people who pose a danger to themselves or others. But I would really like there to be a clear boundary, and what frustrates me is that this boundary doesn't actually exist. When people are unwell, the staff working with them, are essentially left exposed. They [patients] can essentially do anything, commit any criminal act, and the police almost always leave us in the cold. The legal system is equally flawed. So, when the police do take something seriously, the case is always dropped, regardless of whether or not there is injury.”*

## 4 Discussion

In this study we explored the motivations and challenges experienced by mental health professionals working on High and Intensive Care wards. Most confessions reflected pride or being impressed, and a smaller portion expressed frustration with High and Intensive Care work. Professionals appear deeply committed to providing high-quality care driven by four key motivations: meaningful engagement, professional agency, collaborative competence and security. This discussion offers a comprehensive analysis of these insights, with attention to their theoretical and practical implications.

First, motivation to work was driven by the deep and meaningful impact that High and Intensive Care work has on individuals. The severe clinical conditions patients cope with, often lead to empathize deeply with those in care, attracting professionals to this work. In literature this empathy is described as ‘the comfort of closeness’ referring to feelings of closeness and affection by mental health professionals for their patients and the emotional reciprocity synonymous with everyday nursing and caring practice (Deacon et al., 2006). We found many similarities with other studies regarding the rewarding nature of mental health care, such as a sense of career pride and the motivation to help people through difficult times — both of which were identified as key factors attracting professionals to the job (Borghmans et al., 2024; Adams et al., 2021). These key factors can be directly leveraged in recruitment strategies.

Second, High and Intensive Care work is not only rewarding, but also inherently complex. This is a characteristic that, for many participants, was a key reason for choosing to work in this setting. Nevertheless, admissions are often perceived as misaligned with the High and Intensive Care admission criteria, which results in frustration. According to Alexander et al. (2015), nurses view acute mental health roles as highly specialized and demanding, requiring opportunities to apply their skills in complex situations and contribute to clinical decision-making. In line with this, our study showed that nurses’ perceptions of being acknowledged and taken seriously, particularly during escalating situations, are closely linked to their sense of professional value and influence within the care process. Nonetheless, according to our findings, existing salary structures inadequately reflect the workload and level of responsibility assumed by High and Intensive Care nurses, particularly in comparison to their psychiatrist counterparts. According to O'Connor et al. (2024) this may lead to frustration, stress, and burnout, ultimately compromising care and increasing turnover. It is essential to establish greater professional recognition, including higher salaries for nurses, increased autonomy over work schedules, and expanded access to training and development opportunities. This should be accompanied by clear admission criteria to preserve the acute character of the High and Intensive Care, a feature that professionals find particularly appealing.

Third, our findings highlight that attention to the team is considered relevant next to attention to the individual professional. A strong, cohesive team is essential in HIC settings and serves as a key source of motivation for participants. This leads to more effective collaboration, and promotes feelings of safety and professional fulfilment. According to Deacon et al. (2006), the dynamic in acute psychiatry, like High and Intensive Care, fosters a collective mindset of ‘being in this together’, in which the ability to rely on one another, both professionally and personally, is essential. Shared achievements reinforce team pride and strengthen professional identity (Borghmans et al., 2024), a sentiment also expressed by participants in our study. Our findings suggest that, although high workloads, staff shortages, and demanding patient care are often seen as challenges, the ability of staff to manage these pressures effectively can actually foster a sense of pride and become a significant source of motivation. Team integrity is nevertheless challenged by high staff turnover, resulting in relatively young, inexperienced, or temporary teams (de Jong et al., 2025). Feeling competent and working within a competent, supportive, enjoyable team are key factors that can help attract and retain staff. To support this, it is recommended that all disciplines receive structured training in the High and Intensive Care model, as well as in the professional competencies it defines as essential for working within these settings. This training should be a requirement, enabling professionals to reinforce each other’s strengths and contribute to safe, recovery-oriented care. In addition, literature emphasizes that diverse expertise and perspectives are key in managing complex care demands and should not be reduced to generalized staffing roles (Rezaei and Saghazadeh, 2022). We recommend active shaping of team composition, through inclusive recruitment and intentional team-building, to strengthen cohesion, build mutual respect, and encourage reflective practice.

Fourth, safety emerged as a fundamental prerequisite for effective staff performance. Interestingly, unlike previous studies that identified patient-initiated violence as a major factor influencing mental health professionals’ intention to leave the profession (Adams et al., 2021), aggression itself was not raised as a source of frustration in the context of High and Intensive Care work in this study. This suggests that a strong, cohesive, and resilient team can buffer the impact of aggression and foster a greater sense of safety. Participants emphasized that psychological safety was closely tied to working within a stable, experienced, and supportive team, where colleagues could rely on one another during challenging situations. To strengthen this, we recommend that management actively foster a healthy relational culture by addressing internal conflicts, providing training in conflict resolution, and promoting team-building initiatives. A sense of psychological safety requires trust, not only within the team, but also in management and key external stakeholders such as the police. Participants expressed concern regarding the inconsistency of police involvement, particularly in situations where legal or behavioral threats persisted despite psychiatric care. These concerns are echoed by mental health organizations internationally (Duggan, 2024); however, empirical research on collaboration between police and mental health services remains limited and warrants further investigation. Potential benefit may be drawn from a UK protocol designed to clarify the role of police in psychiatric intensive care units (College of Policing, 2021). This model, grounded in clear guidelines and joint case evaluations, may offer a valuable framework for improving inter-agency collaboration, provided it is adapted to national legal and healthcare systems. Moreover, our findings call for increased attention and stronger alignment between mental health services and the legal system, particularly in responding to workplace aggression, emphasizing the need for more decisive action from legal authorities.

Finally, our findings highlight a broader systemic issue: understaffing undermines safe care delivery, which in turn undermines staffing, leading to what Baker et al. (2019) called ‘the vicious cycle of unsafestaffing’. This compromises continuity, dilutes policy implementation, and jeopardizes adherence to the High and Intensive Care model. Structural changes are urgently needed, as many of the motivations and challenges identified stem directly from staffing issues. Without sufficient and well-supported personnel, improvements in care quality and safety will remain out of reach. With persistent staffing shortages suggested to nearly triple within eight years (AZW, 2024) it is highly important that action is taken to counter these shortages. Although mental health nursing is widely seen as meaningful and rewarding, as confirmed by our study, many nurses experience growing emotional exhaustion, moral distress, and burnout (Adams et al., 2021; Ward, 2011). Common contributing factors are intense emotional involvement, exposure to violence, involuntary detentions, underfunding of services, and high rates of mental health issues among staff themselves including suicidal risk (Johnson et al., 2018). And this does not only concern nurses, as a Dutch study reported that nearly half of the psychiatrists cope with emotional exhaustion, moderate to high stress levels, 20% having experienced burnout, and 43% undergoing treatment for mental health issues (Musterd, 2024). Given the severe shortage of psychiatrists in the Netherlands, this is a significant concern. Although we did not examine health impacts in our study, burnout is tightly linked to reduced well-being—a multifaceted construct encompassing mental and physical health and stress management capacity (Johnson et al., 2018). Recruitment and retention efforts must address these realities as well, prioritizing well-being, stress mitigation, job satisfaction, wage parity, safety, educational opportunities, and managerial support (de Vries et al., 2023); all of which are consistent with the findings in this study. Importantly, any interventions should be strategically aligned not only with workforce attrition drivers but also intention to leave. Investing in the recruitment of qualified staff for High and Intensive Care wards should be accompanied by targeted training programs. Providing education not only enhances professional competence but also makes the position more attractive to potential employees.

### 4.1 Strengths and limitations

There are several strengths and limitations. First, we found several strengths to the method of the confession booth. The confession booth may have lowered the chances of social desirability bias (Hecker and Kalpokas, 2025). Though we do not have evidence for this, we believe that our prompts encouraged participants to share authentic, context-specific work experiences rather than generic responses more commonly seen with standard questionnaires. By ensuring a confidential environment and de-identification of the voice messages we invited participants to freely share their experiences. Although not applicable in our study, we can imagine that the confession booth may offer potential in addressing certain methodological challenges, such as engaging hard-to-reach populations, exploring sensitive topics, or working within limited research resources. This potential warrants further exploration. In addition to these practical advantages, the approach also contributes to the development of more creative and accessible research designs. Many participants found the confesson booth particularly appealing due to its innovative nature, a promise that is further supported by literature on creative qualitative methods (Ash et al., 2024). The equal distribution of participation across different shifts supports our idea that allowing individuals to confess at their own convenience is beneficial for settings with shift work. On the other hand, there are some drawbacks to this method. Given the absence of a researcher in the room, there was no opportunity to probe further into an individual's responses. Consequently, we may have missed crucial context or underlying meaning, something we believe may indeed have occurred at times. We had anticipated more depth in participants’ reflections, particularly regarding what truly motivates or drives them in their work, beyond the examples they provided. Following the evaluation, participants were excited and curious what the ‘confession booth’ entailed. However, some had the feeling that using the word ‘confessions’ may have scared people off, as if they had done something wrong. Participants also thought that having to use their own phone may have given privacy concerns. Since confessions were de-identified, we could not distinguish motivations for certain participant characteristics, for example profession, age or experience. In this study we did not include staff who resigned from their positions, potentially omitting more critical yet valuable insights into what could be improved to retain staff.

## 5 Conclusion

Working in High and Intensive Care wards is both meaningful and challenging. Professionals are driven by the opportunity to make real impact, apply clinical expertise, and work within strong resilient teams—provided there is a sense of safety and professional recognition. However, unclear admission criteria, insufficient autonomy, and wage disparities undermine motivation and contribute to frustration. Team cohesion emerged as a source of resilience and decisive factor to shaping staff’s sense of safety. In strong teams, incidents of aggression were perceived as more manageable and did not necessarily undermine psychological safety. However, this cohesion is under pressure due to high turnover and persistent staff shortages. Moreover, inconsistent cooperation of police and lack of clear frameworks hamper the handling of complex and potentially unsafe situations. To maintain the core principles of the High and Intensive Care model, structural investment in staff is urgently needed. Without sufficient and supported staff, core values such as continuity, safety, and non-coercive care cannot be upheld. Recruitment and retention efforts must therefore align with professionals’ needs and realities. Finally, sustainable solutions require commitment at all levels, from policy to practice, to foster a future-proof care environment.

## Multiple publications from a single study

This study was conducted within the context of a broader research project on the High and Intensive Care model and its ongoing development. While the participating institutions also contribute to the larger research initiative, the data presented here were collected specifically for this study and will not be disseminated elsewhere. To date, one article from the broader research project has been published.

## Submission declaration

The article is not under consideration for publication elsewhere. The article's publication is approved by all authors and by the responsible authorities where the work was carried out.

## Funding

This work was supported by the participating mental health care institutions.

## Declaration of generative AI and AI-assisted technologies in the writing process

During the preparation of this work the authors used Microsoft 365 Copilot Chat to improve the readability and language of the manuscript. After using this tool/service, the authors reviewed and edited the content as needed and take full responsibility for the content of this published article.

## Data availability statement

Research data for this article*

Due to the sensitive nature of the questions asked in this study, survey respondents were assured raw data would remain confidential and would not be shared.

*Data not available / The data that has been used is confidential*

*Exploring work motivation in High and Intensive Care wards with the confession booth: an innovative approach

## CRediT authorship contribution statement

**Isa C. de Jong:** Conceptualization, Data curation, Formal analysis, Investigation, Methodology, Project administration, Resources, Validation, Visualization, Writing – original draft, Writing – review & editing. **Laura A. van Melle:** Conceptualization, Formal analysis, Funding acquisition, Methodology, Supervision, Writing – original draft, Writing – review & editing. **Cornelis L. Mulder:** Conceptualization, Methodology, Supervision, Writing – review & editing. **Mariëtte A. van den Hoven:** Conceptualization, Methodology, Supervision, Writing – review & editing. **Yolande Voskes:** Conceptualization, Formal analysis, Funding acquisition, Methodology, Resources, Supervision, Writing – original draft, Writing – review & editing.

## Declaration of competing interest

The authors declare the following financial interests/personal relationships which may be considered as potential competing interests: Laura A. van Melle reports a relationship with GGZ inGeest that includes: employment. Yolande Voskes reports a relationship with Mental Healthcare Centre GGZ Eindhoven that includes: employment. The other authors declare that they have no known competing financial interests or personal relationships that could have appeared to influence the work reported in this paper.

## Appendix A Glossary

**High and Intensive Care:** High and Intensive Care is a care model for acute inpatient care units designed for patients experiencing a severe psychiatric crisis who require a secure (closed) admission setting.

**Care ethics**: The theory of care ethics focuses on contact and the relationship between the persons providing and receiving care.

**Recovery oriented care**: Recovery oriented care stands for treatment which is aimed at the four pillars of recovery: recovery of health, daily functioning, social roles and identity.

**High-Security Room:** The High-Security Room is a confined area with private sanitation and smart home technology to maximize the patient’s control over their surroundings, in which continuous contact with health care professionals is maintained. It is used as last resort for emergency situations unmanageable outside the high-security room. Its use constitutes a coercive intervention that must be continuously supervised and applied for the shortest time possible

**Intensive Care Unit**: The Intensive Care Unit is a unit on the High and Intensive Care ward that consists of large single-patient rooms where patients can receive one-to-one care.

**First five minutes method:** The first five minutes method is an intervention that focuses on how to receive and welcome a patient to the ward, on how to establish contact from the first moment a patient enters the ward and on being attentive to the patient’s needs.

**Compulsory Mental Health Care Act:** The Compulsory Mental Health Care Act is a Dutch law that regulates how and when individuals with a mental disorder can receive mandatory care.

**Confession booth:** A confession booth in research refers to a method where participants can express their thoughts, feelings, or experiences in an enclosed space without direct interaction or influence from the researcher or other participants.

## Appendix B Supplementary materials

Image, application 1

Image, application 2
