# Supplementary material for: Exploring work motivation in High and Intensive Care wards with the confession booth: An innovative approach
Source: Int J Nurs Stud Adv. 2026 Apr 16;10:100530. doi: 10.1016/j.ijnsa.2026.100530 (PMC13101622; doi:10.1016/j.ijnsa.2026.100530)
Supplement: Supplementary file 1 [file mmc1.docx]

# Data availability statement

**Research data for this article***

Due to the sensitive nature of the questions asked in this study, survey respondents were assured raw data would remain confidential and would not be shared.

*Data not available / The data that has been used is confidential*

**Exploring work motivation in High and Intensive Care wards with the confession booth: an innovative approach*
